# Commentary: Cognitive reflection vs. calculation in decision making

**DOI:** 10.3389/fpsyg.2015.00936

**Published:** 2015-07-03

**Authors:** Antonio Mastrogiorgio

**Affiliations:** Interuniversity Research Centre on Sustainable DevelopmentRome, Italy

**Keywords:** numerals, task format, cognitive reflection test, numeracy, ecological rationality

The article of Sinayev and Peters (2015) proposes extensive and experimentally-grounded arguments able to shed light on the debate which compares (i) the hypothesis that Cognitive Reflection mirrors the human ability of suppressing automatic answers in favor of deliberate ones, with (ii) the hypothesis that numerical ability alone is able to predict superior decision making and to account for Cognitive Reflection Test (CRT) (Frederick, 2005) results.

Being composed by numerical tasks, both CRT and Numeracy Tests (involved in Authors' speculation) assume that individuals posses some skills, more or less developed, in representing and processing numerical magnitudes. The nature of such skills is a classical topic of mathematical cognition debate, a topic that in more recent years has been framed in terms of the tension between the general hypothesis that numerical representation is abstract vs. the hypothesis that numerical representation is not abstract and format dependent (see Cohen Kadosh and Walsh, 2009 and the related debate on *Behavioral* & *Brain Sciences*, debate in which one of the two Authors took part).

This tension—abstract vs. format-dependent numerical representation—addresses the question of how individuals map abstract numbers onto magnitude representations and process them. Such debate highlights that *numerical format is not neutral* for the cognitive representation of magnitudes, so it weakens the original hypothesis that numerical processing relies on modality-independent, abstract representation (Cohen Kadosh and Walsh, 2009). Format dependence in numerical processing is crucial if considered from the general hypothesis that mathematical cognition is *embodied*. Within this perspective whether numbers are properly abstract entities, numerals are their “embodiments” within a numerical format. For example the magnitude “4” can be represented by means of different numerals such as OOOO in a graphical format, 4 in Arabic format, 100 in Binary format, IV in Roman format.

The emphasis on format dependence offers relevant insights for Cognitive Reflection vs. Calculation debate, insights that seem to be neglected by the Authors, although similar arguments have been partially addressed in literature (see Peters et al., 2008; Mastrogiorgio and Petracca, 2014).

If numerical format is not neutral but constitutively affects numbers representation then we can reach the speculative consideration that format dependence alone could account for part of the variability of observable: *That numerical format is not neutral implies that the results of the tests (both CRT and Numeracy Tests) could be not invariant under a format change*. Put in other words, the same task (both CRT and Numeracy Test items) could be solved in different manners depending on its format, despite the individual differences in numeracy: For example the way in which similar individuals (equivalent in numeracy) face the well-known “bat and ball problem” (in CRT) or an item of a Numeracy Test could be systematically different if the magnitudes are expressed, say, by graphical representations or Arabic numerals. From this perspective the format of a task matters because it triggers a specific reasoning process and leads to a specific solution (see Kotovsky et al., 1985). The consequence is relevant: An individual with higher numeracy could obtain a inferior performance just because the task is implemented using a specific format, and vice versa.

The theoretical implications of non-neutrality of task format are two:

1. *The format is part of the content of a task*. Real world decisions are always “embodied” in specific formats, hence the role of the numerical format is not just a matter of accuracy of magnitudes representation, but it is a matter of how real-world task environments are structured. Whether laboratory settings are able to neutralize the role of different numerical format, real-world decisions always occurs within a format; and such decisions occur only one time and there is no second chance to control the eventual bias in representation, due to the format. For example the epistemic community of technical traders analyzes quotations—and make decisions—by means of charts instead of, say, spreadsheets filled with Arabic numerals.
2. *The format of a task enables a specific solution of the task*. A relevant literature on heuristic decision making casts light on how specific tasks are faced by means of specific heuristics, in particular individuals use heuristics based on the use of prominent numbers for decomposing numerical stimuli in the decimal format (see Gigerenzer and Selten, 2002). In this perspective the format is part of the solving process. For example the performance of technical traders is strictly related to the fact that they heuristically try to predict quotations by means of specific graphical signals instead of calculating with Arabic numerals.

In conclusion, placing emphasis on the non-neutrality of task format, we solicit to reconsider Cognitive Reflection vs. Calculation debate on the footsteps of Simon's legacy, relying on the consideration—known as “scissors” argument—that reasoning abilities are a matter of adaptation to environmental demands. We should be aware of the limits of evaluating decision performance just as a matter of individual cognition-bounded, abstract reasoning abilities (such as CRT or Numeracy Tests assume). And we should be aware that evaluating reasoning abilities requires the ecological caveat that tasks are “embodied” in specific, real-world environments.

## Conflict of interest statement

The author declares that the research was conducted in the absence of any commercial or financial relationships that could be construed as a potential conflict of interest.
